# Combining the advantages of 3-D and 2-D templating of total hip arthroplasty using a new tin-filtered ultra-low-dose CT of the hip with comparable radiation dose to conventional radiographs

**DOI:** 10.1007/s00402-022-04697-7

**Published:** 2022-12-03

**Authors:** Dominik Kaiser, Armando Hoch, Stefan Rahm, Christoph Stern, Reto Sutter, Patrick O. Zingg

**Affiliations:** 1 Department of Orthopaedics, Balgrist University Hospital, University of Zurich, Forchstrasse 340, 8008 Zurich, Switzerland; 2 Department of Radiology, Balgrist University Hospital, University of Zurich, Zurich, Switzerland

**Keywords:** Total hip arthroplasty, Tin-filtered ultra-low-dose CT, THA, Templating, Magnification error

## Abstract

**Background:**

Inaccurately scaled radiographs for total hip arthroplasty (THA) templating are a source of error not recognizable to the surgeon and may lead to inaccurate reconstruction and thus revision surgery or litigation. Planning based on computed tomography (CT) scans is more accurate but associated with higher radiation exposure. The aim of this study was (1) to retrospectively assess the scaling deviation of pelvic radiographs; (2) to prospectively assess the feasibility and the radiation dose of THA templating on radiograph-like images reconstructed from a tin-filtered ultra-low-dose CT dataset.

**Methods:**

120 consecutive patients were retrospectively analyzed to assess the magnification error of our current THA templates. 27 consecutive patients were prospectively enrolled and a radiographic work-up in the supine position including a new tin-filtered ultra-low-dose CT scan protocol was obtained. THA was templated on both images. Radiation dose was calculated.

**Results:**

Scaling deviations between preoperative radiographs and CT of ≥ 5% were seen in 25% of the 120 retrospectively analyzed patients. Between the two templates trochanter tip distance differed significantly (Δ2.4 mm, 0–7 mm, p = 0.035)), predicted femoral shaft size/cup size was the same in 45%/41%. The radiation dose of the CT (0.58 mSv, range 0.53–0.64) was remarkably low.

**Conclusion:**

Scaling deviations of pelvic radiographs for templating THA may lead to planning errors of ≥ 3 mm in 25% and ≥ 6 mm in 2% of the patients. 2-D templating on radiograph-like images based on tin-filtered ultra-low-dose CT eliminates this source of error without increased radiation dose.

**Level of evidence:**

Retrospective and prospective comparative study, Level III.

## Introduction

Freedom of pain, excellent function and long-term survival are expected of total hip arthroplasty (THA) by the patient. A physiological reconstruction of leg length and offset as well as correct sizing and positioning of the components are crucial [1–11]. Leg length discrepancy (LLD) after THA is a common problem: a marked LLD may lead to substantial disability, revision surgery and is the most common reason for litigation against orthopedic surgeons [12, 13]. A LLD of more than ± 5 mm is generally perceived by the patient and may lead to hip pain (hip abductors and flexors) [14–16] or predispose to instability and hip abductor insufficiency both leading to a significantly lower oxford hip score [17]. Under-sizing of the femoral shaft may lead to early subsidence while oversizing may increase the risk of intraoperative femoral fractures.

The best conditions to restore physiological anatomy and leg length are established by precise preoperative planning [18–20]. Traditional analog or digital planning methods ultimately rely on accurately scaled radiographs.

**Fig. 1:**
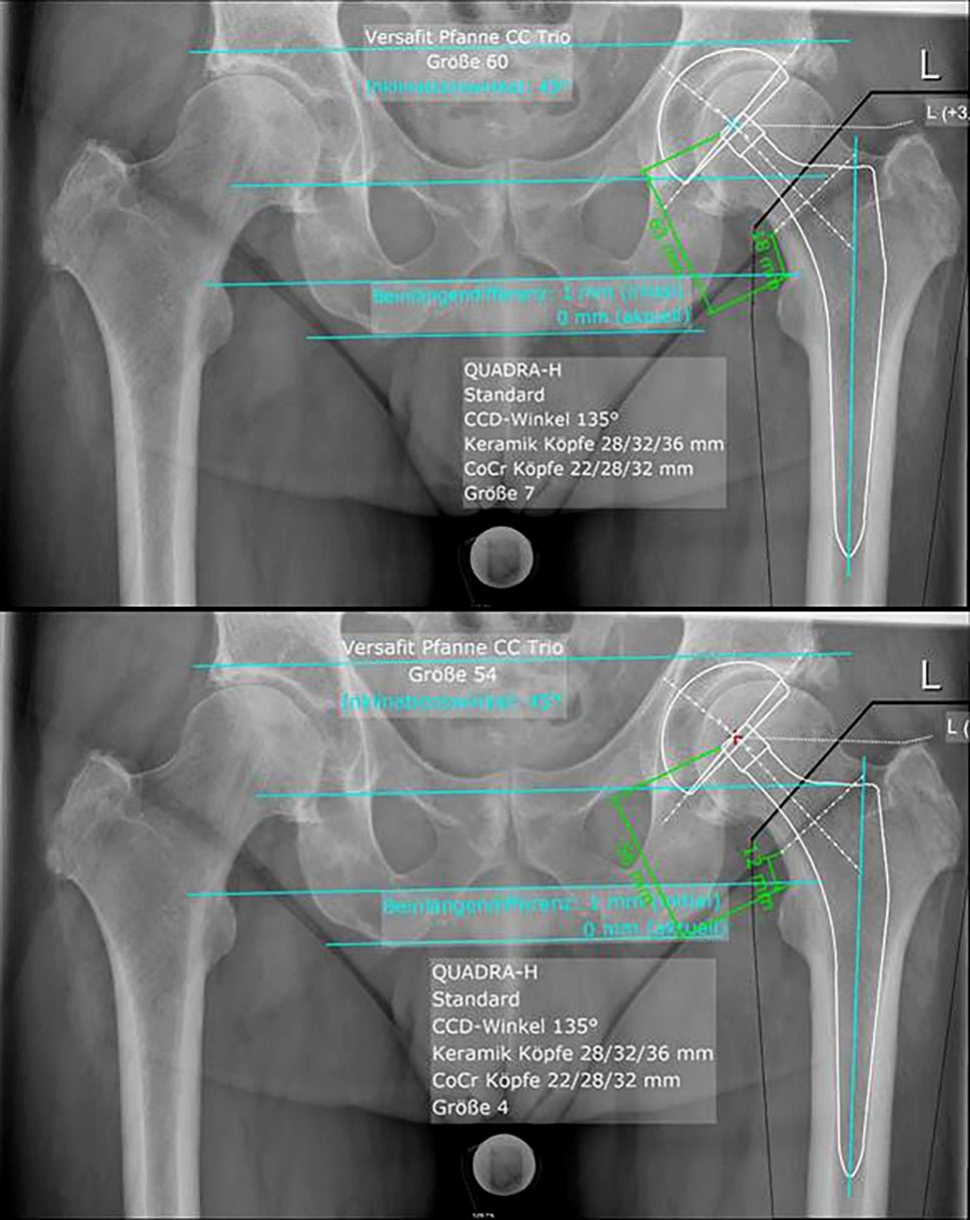
Anteroposterior pelvic radiograph of the same patient scaled with the metal ball 25 mm (top) and the head diameter of the contralateral femoral head obtained from the CT data (bottom) (scaling difference of 11%). Note the distinct difference of the trochanter tip distance of 63 mm (top) to 56 mm (bottom) and the difference in prosthetic component size for both the acetabular cup (60 mm (top) and 54 mm (bottom)) and the stem (size 7 (top) and size 4 (bottom). This scaling difference cannot be detected preoperatively

Several scaling methods exist all relying on the radiology assistant to position the calibration marker as close as possible to the plane of the hip joint [21–23]. Incorrect placement of the calibration marker leads to magnification errors [24] and thus to planning inaccuracies (Fig. 1).

To avoid inaccuracies due to scaling errors, templating on CT image data and bi-planar radiographs (EOS) have been investigated. Both systems are based on parallel radiographs eliminating size distortion. Three-dimensional planning on CT scans has shown a minimal discrepancy between planned and achieved leg length of 0.3 mm (Standard deviation (SD): 2.3 mm, range − 5 to 6 mm) an exact planning of the overall offset, which is often underestimated on plain radiograph [25–27], and a greater accuracy regarding prediction of component size compared to 2-D planning [28, 29]. A more accurate prediction of stem size compared to 2-D planning has also been shown on bi-planar radiographs [30, 31].

In summary, planning on CT is desirable, however, the greater radiation dose of 1.5–4.0 mSv [29, 32, 33] even for contemporary low-dose CT imaging remains a major disadvantage especially in younger patients [34] as it is associated with a 5–17 × increased relative risk of malignancy. Additional disadvantages include the lesser availability and the higher cost of approximately 250$ for a CT compared to 125$ for the radiographs.

We created a tin-filtered ultra-low-dose CT scan protocol (C.S., R.S.) to obtain the necessary images in the exact same position as the pelvic anteroposterior (ap) radiograph without the risk of scaling inaccuracies with additional information including femoral torsion, femoral offset and the extent of the anterior and posterior acetabular wall. All factors that can distort the image, such as pelvic tilt, malrotation of the pelvis or femur, can be detected by the surgeon and thus the examination is more independent of the examiner.

With the implementation of a tin-filter for the latest 3rd-generation CT scanners and spectral shaping of the radiograph beam, a remarkable reduction of radiation dose for pelvic images has been achieved with an average radiation dose of 0.38 mSv [35]. By reducing the radiation dose of the CT scan to levels comparable to the radiograph, the main reason for not routinely performing a CT scan for THA templating is eliminated.

Thus, the aim of this current study was to:

1. retrospectively objectify the relative and absolute scaling deviation of our current planning basis, i.e., pelvic ap radiograph to CT.
2. prospectively assess the feasibility and the radiation dose of THA templating on radiograph-like images of the tin-filtered ultra-low-dose CT data.

## Materials and methods

### Ethical approval

Ethical approval was obtained at the local ethics committee (KEK ZH: BASEC 2021-01956).

#### Part 1: Assessment of the scaling deviation of the current planning basis

A retrospective analysis of a consecutive series of 133 patients was performed. All of these patients received a unilateral primary THA and had a radiologic work-up including a pre- and post-operative radiograph with a 25 mm scaling ball and a preoperative CT scan suitable for 3-D THA templating. To measure the influence of the magnification, factor discrepancy between the images the first author (D.K.) measured the contralateral femoral head diameter as the most constant anatomic landmark present in all images. Due to femoral head deformity, thirteen patients were excluded as a reliable measurement was not feasible, thus including 120 consecutive patients. The diameter of the contralateral femoral head was measured on the radiograph in the MEDICAD planning software (MediCad Multimedia Co., Niedernviehbach, Germany) after scaling the preoperative image with the 25 mm ball and on the post-operative radiograph scaled with the 25 mm ball as well as the known prosthetic femoral head. The same contralateral femoral head diameter was also measured on the coronal CT scan image where the femoral head diameter was largest using the institutional PACS program (Phönix PACS GmbH, Freiburg im Breisgau, Germany) as this most closely matches the summation effect of the radiograph. The differences in the femoral head diameter between the different image modalities as well as the different scaling factors were noted and compared.

#### Part 2: Feasibility of THA templating on radiograph-like images and assessment of radiation dose of tin-filtered ultra-low-dose CT

A prospective series of 27 consecutive patients (29 THA) scheduled for primary cement-less THA were evaluated. Patients under 18 years old and/or with contralateral THA were excluded as in clinical practice an image calibration via the contralateral prosthetic head would be possible. Patient demographics were obtained from the chart (sex, age, body mass index (BMI)).

In our institution, we obtain ap pelvic radiograph in supine position so that the patella faces upwards with a standardized film/focus distance of 120 cm and centered between symphysis and anterior superior iliac spine. Templating is performed by MEDICAD (MediCad Multimedia Co., Niedernviehbach, Germany) and scaling of the image is done by a 25 mm metal ball. The metal ball is placed beside the hip or between the legs by the radiology assistant as close as possible to the plane of the hip joint by palpating the greater trochanter.

**Fig. 2:**
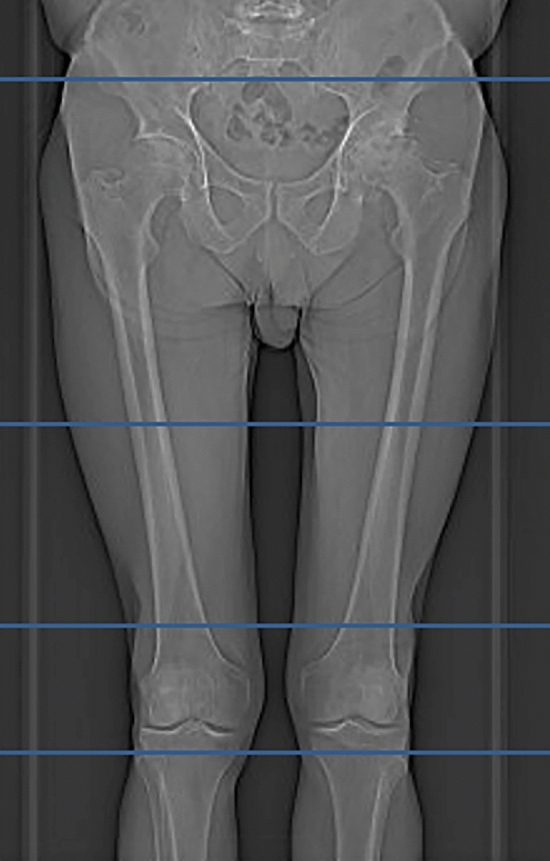
Image depicting the scout of the CT scan protocol. The scanned regions are bordered by the blue boxes and include the hip joint the proximal femur and the distal femoral condyle

In addition to our standard radiographic work-up (pelvic ap view and unilateral cross-table lateral view), a tin-filtered ultra-low-dose CT (protocol Sn 140 kV/150 mAs) was performed on a 128-slice-CT scanner (SOMATOM Edge Plus, Siemens Healthineers). The scan region included the hip joint (3 cm above the acetabular roof) and the 1st half of the femur (11 cm below the lesser trochanter). An additional short scan of the distal femoral condyle was obtained for femoral torsion measurement as this is of clinical interest (Fig. 2).

The CT images were obtained in the same supine position as the pelvic radiograph and the CT images in part 1. A multiplane reconstruction of the obtained CT images with 0.5 mm slice thickness was performed in the institutional PACS program (Phönix PACS GmbH, Freiburg im Breisgau, Germany). The coronal images of interest were summed up and using the mean mode, a radiograph-like image comparable to a pelvic ap radiograph was calculated. This process requires approximately 20 s per patient. Preoperative templating of the THA was performed on the regular pelvic ap radiograph and the radiograph-like image using MEDICAD planning software by the first author four weeks apart. The planned trochanter tip distance (distance from most proximal part of lesser trochanter to medial tip of prosthetic cone) as well as the prosthetic component size was noted and compared.

The radiation dose of the tin-filtered ultra-low-dose CT of the pelvis/proximal femur and distal femoral condyle as well as of the pelvic ap and cross-table lateral radiograph was calculated and compared for each patient. Dose parameters of the CT and the radiograph were extracted from the dose report. Effective CT dose in millisievert was estimated by multiplying the dose length product (DLP) with a standard conversion factor *k*. For *k*, we used a weighted average of the value for the adult hip (0.011 mSv/mGy*cm) and the knee (0.0004 mSv/mGy*cm) [36]. For dose estimation of radiograph, the dose area product was multiplied with *k* = 0.00029 mSv/mGy*cm^2^ [37].

### Statistical analysis

Power analysis of part 1 revealed that 44 patients are necessary to reveal a difference of 2% ± 4% deviation with a power of 0.9 and an *α*-error of 0.05; of part 2 revealed that 26 patients are necessary to reveal a difference of 0.1 mSv ± 0.15 mSv with a power of 0.9 and an *α*-error of 0.05. Statistical analysis to determine the difference between the contralateral femoral head diameter, the difference in trochanter tip distance and the difference in radiation dose between the tin-filtered ultra-low-dose CT and the radiographs were performed using the paired student’s *t*-test. Differences were considered to be statistically significant for *p *values < 0.05. Results are reported as means, standard deviation (SD), range and associated *p *values if not stated otherwise.

## Results

### Part 1: Assessment of the scaling deviation of the current planning basis

**Fig. 3:**
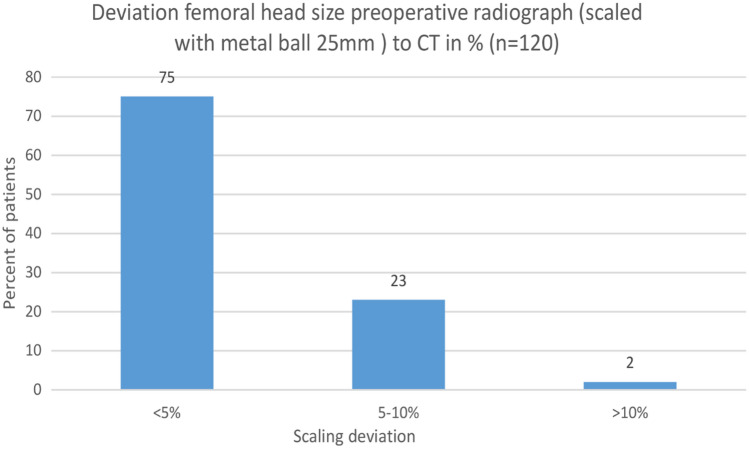
Graph depicting the percentual deviation of the contralateral femoral head diameter between the preoperative radiograph scaled with the metal ball (25 mm) and the preoperative CT

A total of 120 consecutive patients with full radiologic work-up were included in the analysis. The mean diameter of the contralateral femoral head was 48 mm (range 40–55 mm) when measured on the post-operative radiograph scaled with the known diameter of the prosthetic head. The mean diameter of the same contralateral femoral head was 49 mm (range 39–59 mm) when measured on the preoperative radiograph scaled with the 25 mm metal ball. The mean percentual deviations are summarized in Table 1, the differences were highly significant (*p* < 0.001). The number of deviations greater than 5% and 10% between the CT and the preoperative radiograph scaled with the 25 mm metal ball is depicted in Fig. 3.

**Table 1:** Mean diameter of the contralateral femoral head (compared to CT)

Image	Scaled with	Mean diameter [mm]	SD [mm]	Range [mm]	deviation to CT [%]	SD	Range	*p* = *
Preoperative radiograph	25 mm metal ball	49	4.1	39–59	3.65	2.62	0–11.48	** < 0.001**
Postoperative radiograph	25 mm metal ball	49	3.9	39–59	3.18	2.28	0–9.45	** < 0.001**
Postoperative radiograph	Prosthetic head	48	3.75	40–55	1.36	1.01	0–4.56	0.8488
CT scan	–	48	3.9	39–56		–	–	–

^*^paired Student’s *t* test

### Part 2: Feasibility of THA templating on radiograph-like images and assessment of radiation dose of tin-filtered ultra-low-dose CT

The results are summarized in Table 2. THA templating was feasible on all radiograph-like images. In the patient with the highest radiation dose (0.74 mSv), we deliberately chose to include the entire pelvis to allow for 3-D planning as well. By excluding this patient, the mean radiation exposure was 0.58 mSv (SD 0.037, range 0.53–0.64). The mean radiation dose of the additionally performed short scan of the distal femoral condyle was 0.01 mSv (SD 0, range 0.01–0.01). The mean radiation dose of the pelvic ap and cross-table lateral radiograph was 0.34 mSv (SD 0.08, range 0.19–0.49) in our patient collective and thus significantly lower than the radiation dose of the tin-filtered ultra-low-dose CT (*p* < 0.001).

**Table 2:** Feasibility of THA templating on radiograph-like images and assessment of radiation dose of tin-filtered ultra-low-dose CT

Age	61y (21–85y)			
BMI	28.2 (19.6–39.2)			
Number of patients/hips	27		29	
Female/male	10		17	
Trochanter tip distance	Radiograph: 56.8 mm (39–70 mm)	Radiograph-like image: 55.8 mm (37–65 mm)		p* = *0.035*
Mean deviation of trochanter tip distance	2.38 mm (0–7 mm)			
Predicted femoral shaft size between radiograph and radiograph-like image	Same: 45% Within ± 1 size: 97%			
Predicted cup size between radiograph and radiograph-like image	Same: 41% Within ± 1 size: 86%			
Radiation dose	Radiograph: 0.34 mSv (0.19–0.49 mSv)	Radiograph-like image: 0.59 mSv (0.53–0.74 mSv)		p* = *0.01*

^*^Significance level *α* ≤ 0.05

## Discussion

**Fig. 4:**
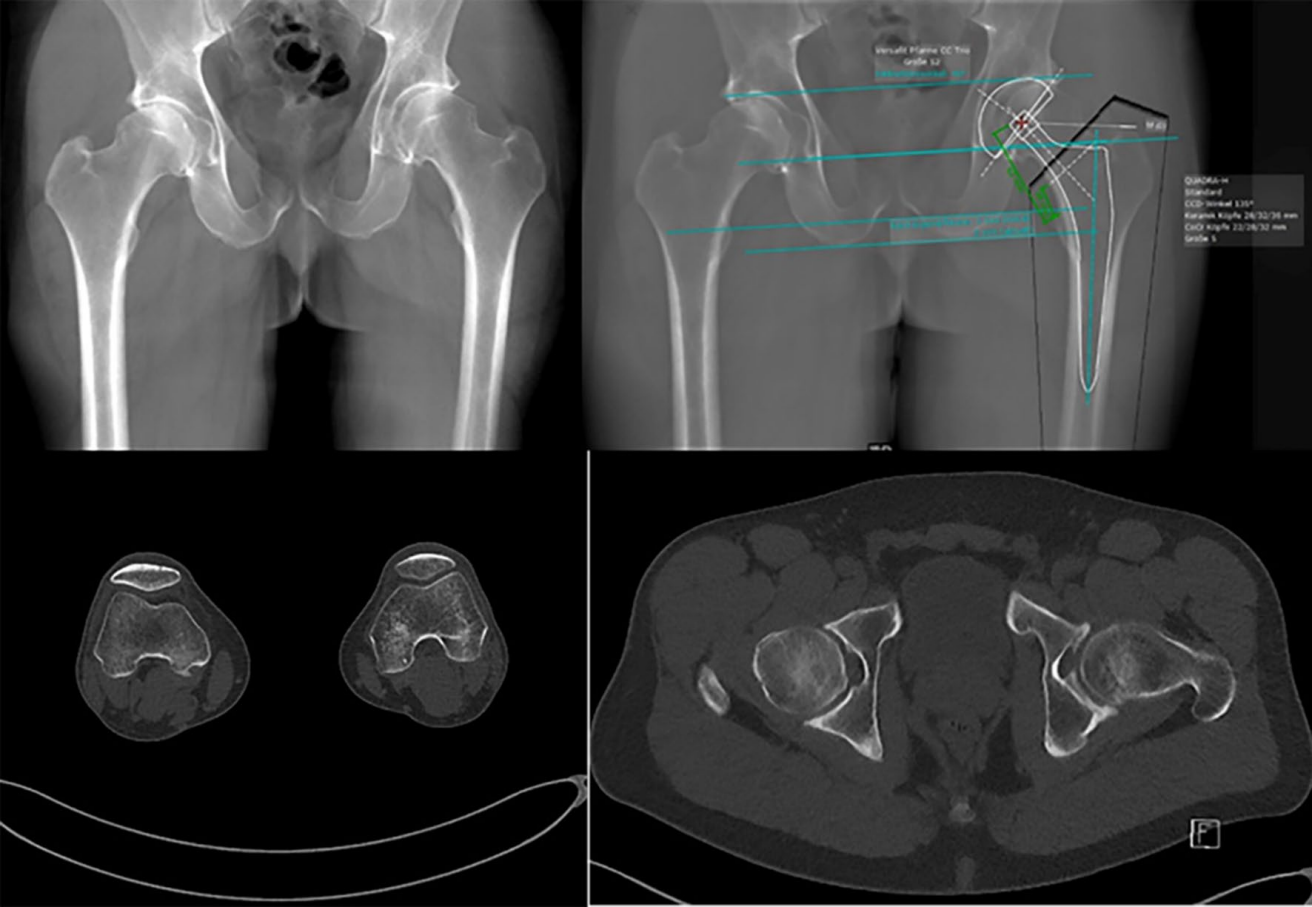
*Top left*: Radiograph-like image reconstructed from CT image data. *Top right*: THA templating performed on radiograph-like image. *Bottom left*: Rotation of femoral condyle. *Bottom right*: Rotation of femoral neck

The two key findings of this study are that (1) the scaling deviation can lead to a planning error regarding the trochanter tip distance of ≥ 3 mm in 25% and ≥ 6 mm in 2% of the patients, (2) the newly developed tin-filtered ultra-low-dose CT scan protocol enables reconstruction of high-quality radiograph-like images which can readily be used for THA templating (Fig. 4, top left) with remarkably low radiation dose.

The mean scaling deviation of our current planning basis for THA is 3.2% (range 0–9.45)–3.7% (range 0–11.48), which is markedly lower than the reported 6.8% (range 0–26%) [24]. We attribute this to the qualifications of our highly specialized musculoskeletal radiology department and the possibly lesser measurement error, as we measured a larger structure [24]. Nonetheless, a deviation of ≥ 5% was seen in 25% of the patients and ≥ 10% in 2% of the patients. The mean trochanter tip distance in our templates was 56.8 mm translating to a difference of ≥ 3 mm in 25% and ≥ 6 mm in 2%. In one patient, a scaling error of 11% occurred leading to a difference in planned trochanter tip distance of 7 mm as seen in Fig. 1, a discrepancy in acetabular cup diameter of 6 mm and a stem size difference of 3.

This scaling deviation may partially explain the lesser accuracy of predicting stem and cup size compared to CT-based planning [28, 29]. This is also reflected in our low rate of agreement regarding the shaft and cup size between the “template on conventional radiograph” and the “template on radiograph-like image” of only 45%/41%.

The greatest risk of scaling errors is that they cannot be detected unlike a malrotated or tilted pelvic ap radiograph. In low volume clinics, an even greater average deviation from reality is conceivable possibly negatively affecting clinical outcome [1–11, 14, 15, 17], revision surgery and litigation rate [12, 13, 16].

Templating on a 2-D radiograph-like image of the pelvis reconstructed from CT image data eliminates this potential magnification error (Fig. 4, top left and right). Other advantages include (a) direct visualization of the femoral offset and torsion and (Fig. 4, bottom) (b) use of familiar and efficient 2-D planning method and (c) verification of correct patient positioning during the CT scan regarding all aspects. In select complex cases, the scan can be extended to include all of the pelvis as well as the ankle and allow 3-D planning with a minimally greater radiation dose (0.74 mSv vs. 0.58 mSv).

In our prospective series, the mean radiation dose of the tin-filtered ultra-low-dose CT protocol was significantly lower than comparable values in the literature for pelvic CT protocols ranging from 1.5 mSv (SD 0.1) to 2.8 mSv (SD 0.8) (*p* < 0.001) [32, 33] and lower than the radiation doses of conventional pelvic and cross-table axial radiographs as reported in the literature (0.3–0.83 mSv) [37–39]. The radiation dose is greater than in the previously published [35] series as we have used a different protocol ((protocol Sn 140 kV/150 mAs)) with a greater energy to account for the expected lower bone density in this older patient collective. The radiation dose of the CT protocol remained significantly higher than the radiation dose of the conventional radiographs performed at our institution (*p* < 0.001) (Table 2). However, this difference is likely smaller in everyday practice as it is not uncommon for conventional radiographs to be re-taken due to inadequate image quality (malrotation, pelvic tilt, centering). In these cases, the full radiation dose must be reapplied, while for CT scans the scout function can prevent unnecessary radiation of the patients. Additionally, by further reducing the number of dissatisfied patients, the need for post-operative imaging to understand the cause of persistent complaints can be decreased. In terms of time requirements for our radiology department, the two modalities are comparable. A disadvantage for the CT remains the lesser availability of the device and the slightly higher cost (250$ vs 125$).

The main limitation of this study is that we do not investigate the influence of this scaling inaccuracy on clinical outcome. Nonetheless, we are convinced that by eradicating this source of error, we can reduce the number of outliers especially regarding leg length discrepancy, a common cause of patient dissatisfaction and litigation [16, 40]. A further limitation is the variance of the measurement technique, albeit using the contralateral femoral head, we have chosen a large clearly defined anatomical structure, thus reducing this error as much as possible.

In summary with this new CT scan protocol, a radiograph-like image of high quality can be readily obtained and used to template THA in the accustomed 2-dimensional manner and if desired easily extended for 3-D planning. Errors due to incorrect scaling are eliminated. Other sources of errors like incorrect patient positioning, incorrect rotation of the femur can be directly excluded by the surgeon himself. Additional information including (a) femoral torsion and offset, which can be measured directly; (b) extent of anterior and posterior wall; (c) exact depiction of osteophytes can be directly visualized in complex cases. All of these advantages can be obtained with a radiation dose significantly lower than values for CT reported in the literature, comparable to values for conventional radiograph in the literature and slightly greater than the radiation dose for conventional radiographs at our institution.

## Conclusion

Scaling deviations of ap pelvic radiographs for templating THA may lead to planning errors of ≥ 3 mm in 25% and ≥ 6 mm in 2% of the patients compared to CT-based planning. 2-D templating on radiograph-like images based on tin-filtered ultra-low-dose CT eliminates this source of error and allows templating in the accustomed efficient manner with an extremely low radiation dose.
